# Anxiety and Depression: A Comparison between Living and Cadaveric Renal Transplant Recipients

**Published:** 2011-11-01

**Authors:** Z. Parsaei Mehr, M. Hami, Z. Moshtagh Eshgh

**Affiliations:** 1 *Faculty of Nursing and Midwifery, Islamic Azad University, Tehran Medical Branch, Tehran, Iran,*; 2 *Nephrology Unit, Department of Internal Medicine, Mashhad University of Medical Sciences*; 3 *Department of Nursing, Faculty of Nursing and Midwifery, Shahid Beheshti University of Medical Sciences*

**Keywords:** Depression, Anxiety, Kidney transplantation

## Abstract

Background: Anxiety and depression are the most common psychological disorders in kidney transplant recipients that may affect disease process and graft survival.

Objective: Based on the types of kidney donation in our country, living vs. cadaveric donation, we conducted this study to compare psychological problems in renal recipients.

Methods: This cross-sectional study was conducted on kidney transplant recipients who were categorized according to their donors to “living” and “cadaveric” groups. Patients with stable condition were followed monthly in outpatient clinics. The psychological status of each patient was assessed by clinical interview and Spielberg State Trait Anxiety Inventory and the Beck Depression Inventory (BDI). The calculated Cronbach alpha for the reliability of the total scale was 0.95.

Results: We recruited 120 recipients (60 patients in each group of living and cadaveric donor transplantation) for the study. There was no significant difference in demographic data between two studied groups (p>0.05). The mean±SD anxiety score was significantly lower among living transplant recipients compared to cadaveric transplant recipients (80.2±15.2 vs. 86.9±18.8 p=0.03). We also found significant relation between depression score and kind of graft donation (11.6±5.7 in living vs. 16.4±9.4 in cadaveric groups; p<0.005).

Conclusion: Psychological problems such as depression and anxiety are significantly higher in cadaveric than living renal recipients. Periodic psychological evaluations should be recommended for kidney transplant recipients, especially for the cadaveric group.

## INTRODUCTION

In patients with end-stage renal disease (ESRD), renal transplantation is the best method of replacement therapy, even from psychological point of view. In hemodialysis patients, depression is very common. This finding is acceptable since depression follows loss, and this group of patients loses their independence, health and energy [1]. After transplantation, some of these limitations are reduced. So transplantation is associated not only with better quality of life and survival, but also reduced medical expenses and mental disorders [2].

On the other hand, transplantation has its own problems some of which (*e.g.*, side effects of drugs such as corticosteroids, fear of rejection, sense of foreign organ) may lead to mental disorders [1].

Anxiety and depression are the most common psychological disorders in kidney transplant recipients [3] that may affect the disease process and graft survival [4]. Anxiety and depression have been associated with noncompliance with medication use and personal care, compromised quality of life, and difficulty in integrating the newly acquired graft into their sense of self [5]. DiMatteo, *et al*, in a meta-analysis reported that depressed patients were threefold more likely to be nonadherenet to the medical regimen than patients without depression [6]. Penkower, *et al*, also suggested that psychological stress such as anxiety and anger may lead to nonadherance to drugs in adolescent renal transplant recipients [7]. There are two types of kidney donation. Considering the conflicted data on the incidence of anxiety and depression in living *vs*. cadaveric renal recipients [2], we conducted this study to compare the incidence of the said disorders in transplant recipients in our center.

## MATERIALS AND METHODS

Participants

This cross-sectional study was conducted in the year 2010 at Ghaem and Imam Reza Hospitals affiliated to Mashhad University of Medical Sciences, Mashhad, northeastern Iran. Following ethical approval, transplant recipients who were treated and followed in our transplant clinics were invited to participate in this study. Inclusion criteria included age between 18 and 65 years, a minimum of six months since transplant operation, tendency to participate, not being concurrently hospitalized or treated for rejection or infection episodes, and fluency in spoken Persian.

Out of 186 patients in clinics, 120 gave consent to enter the study (response rates 64.5%). The recruited sample consisted of 86 (71.7%) men, and 34 (28.3%) women. The mean±SD age in all patients was 37.3±9.9 years. Then, based on the type of donation, they were divided into “living” and “cadaveric” groups (60 people in each group).

Measurements

At first, in both groups, participants’ medical records were reviewed to obtain information about previous dialysis and its duration and type, transplant history, type of donor, education level, their job and marital status, and re-transplantation history.

Symptoms of depression were assessed with the Beck Depression Inventory II (BDI). BDI includes 21 multiple choice items that describe specific depressive symptoms over the past week.

This instrument has been administered extensively and shown to have good reliability coefficients ranging from 0.73 to 0.92. While scores on the BDI II of 0-9 are considered normal, scores of ≥10 indicate that the patient is symptomatic. Scores between 10 and 18 are considered mild depression. Moderate depression is diagnosed, if BDI score was between 19 and 29. Scores ≥30 is considered severe depression.

Anxiety symptoms were assessed with the State Anxiety subscale of the Spielberger State-Trait Anxiety Scale. The State anxiety is characterized by subject’s subjective feelings of tension, apprehension, nervousness and worries, and by activation or arousal of the autonomic nervous system. This 40-item instrument was administered to all patients. Each item is scored on four levels of anxiety intensity from 1 (not at all) to 4 (very much). The total score is between 40 and 160. Estimates of internal consistency was determined by Cronbach’s alpha for the State anxiety that exceeded 0.95. 

Statistical analysis

Statistical analysis was conducted using the SPSS ver 13.0 (SPSS Inc, Chicago, IL, USA). The data are expressed as mean±SD. Univariate relationships between independent variables (anxiety, depression) and type of donors, were assessed by independent sample *Student’s t test. *Fisher’s exact test was used to assess the impact of demographic variables on severity of psychological problems in each group of recipients. A p<0.05 was considered statistically significant.

## RESULTS

Sociodemographic and medical characteristics of the two studied groups of cadaveric *vs*. unrelated living donors are summarized in Table 1. There was no significant difference between demographic data in the two groups. The two groups were not significantly different in terms of education level. Most of the patients had undergraduate level in both cadaveric group (n=51, 85%) and living donor group (n=43, 71.7%). The number of graduated patients was higher in living group (n=13, 21.7%) but the difference was not significant. The mean±SD time spent on dialysis prior to transplantation was significantly (p<0.001) higher for cadaveric group patients (36.2±18.3 months) than living transplant patients (22.5± 21.1 months).

**Table 1:** Demographic data of subjects in our study

**Parameter**	**Living Donors**	**Cadaveric Donors**	**p value**
Sex (M/F)	41/19	45/15	0.6
Mean±SD age (yr)	37±9.5	37±10.2	0.7
Dialysis (HD/PD)	51/9	56/4	0.1
Employed/Unemployed	27/33	22/38	0.4
Married/Single	53/7	44/16	0.06
Hx of rejection	10 (17%)	7 (12%)	0.3
Hx of re-transplantation	11 (18%)	12 (20%)	0.5
Mean±SD serum creatinine (mg/dL)	1.3±0.4	1.4±0.4	0.4
Hx of hospitalization	16 (27%)	22 (37%)	0.2
Time since transplant(months)	53.8±16.9	22.1±16.9	<0.001

The mean±SD anxiety score was significantly lower among living than cadaveric transplant recipients (80.2±15.2 *vs*. 86.9±18.8, p=0.03). While most of the living recipients had mild anxiety, most of the cadaveric recipients had moderate anxiety (p<0.05, Table 2). 

**Table 2:** Incidence of anxiety in renal transplant recipients

**Anxiety Severity (score)**	**Living Donors**	**Cadaveric Donors**
Mild (40–79)	35 (58%)	25 (42%)
Moderate (80–119)	23 (39%)	33 (55%)
Severe (120–160)	2 (3%)	2 (3%)
Total	60 (100%)	60 (100%)
p<0.005		

There was also significant association between depression score and type of graft donation; the mean±SD depression score was 11.6±5.7 in living *vs*. 16.4±9.4 in cadaveric donor groups (p<0.005). The prevalence of depression was also significantly higher in cadaveric group (62%) than living donor group (52%) (Table 3). 

**Table 3:** Incidence of depression in renal transplant recipients

**Depression Severity (score)**	**Living Donors**	**Cadaveric Donors**
None (0–10)	29(48%)	23(38%)
Mild (11–18)	22(37%)	15(25%)
Moderate (19–29)	9(15%)	14(23%)
Severe (>30)	0	8(13%)
P<0.001		

Although analysis of variances revealed a significant association between age and sex of patients in both cadaveric and living donors with anxiety and depression, in other variables such as job, marital status, education level, duration of dialysis (only in group with depression), and history of re-transplantation, we did not find any relation (Table 4).

**Table 4:** Relation between studied variables and anxiety and depression scores in the two studied groups

**Variable**	**Mean±SD anxiety score**		**Mean±Sd depression score**	
	**Living donors**	**Cadaveric donors**	**Living donors**	**Cadaveric donors**
Sex				
female	79.8±15.5	104.5±12.3	11.9±5.4	24.6±8.6
male	78.2±12.1	78.7±15.3	11.5±6.01	12.6±7.03
p value	0.005		0.005	
Age(yr)				
<35	82.0±11.6	82.2±16.7	11.8±4.7	16.1±6.4
>35	77.1±11.3	92.0±17.2	12.0±6.8	16.9±6.9
p value	0.01		0.002	
Dialysis duration (months)				
<35	77.3±12.0	87.6±11.4	11.2±6.8	16±7.7
>35	75.5±10.0	86.1±10.7	11.1±5.4	14.4±7
p value	0.07		0.001	
Job status				
employed	13.2±79.3	9.6±90.1	6.6±12.3	9.6±16.6
unemployed	12.4±78.1	9.4±85.1	5.4±11.2	9.3±16.2
p value	0.8		0.5	
Marital status				
single	6.7±78.2	9.1±81.7	5.4±12.1	1.9±15.6.6
married	7.8±78.8	7.7±88.9	5.9±11.6	1.6±16.6
p value	0.4		0.7	
History of re-transplantation				
yes	6.2±78.4	10.8±85.1	5.3±10.3	8.8±14.2
no	7.9±78.3	9.1±87.4	6.2±11.9	9.5±16.9
p value	0.9		0.7	

## DISCUSSION

It is known that anxiety and depression are more common among kidney transplant recipients than in healthy controls [6]. We found that 15% of living kidney recipients and 37% of living group recipients had moderate to severe depression. In other studies, the prevalence of depression in transplanted patients varies from 20% to 75% [9-11].

We found that 58% of cadaveric group recipients and 42% of living group recipients had moderate to severe anxiety. So anxiety also had significantly higher prevalence in cadaveric group (p<0.005). Some previous studies reported similar results; they found that about half of their patients suffered from anxiety even years after successful transplantation [12-13]. None of the studies report any difference in the type of transplantation in terms of the type of donors (living *vs*. cadaveric). However, the reported means were totally comparable with our findings.

Prevalence of depressive and anxious symptoms was lower in men. However, in healthy population depression and anxiety are more common among women than men [7]. This is in contrast to findings of Penkower, *et al*, who have shown that the distribution of depressive and anxiety symptoms among boys and girls were similar [7].

In our series, patients older than 35 years had more severe forms of depression and anxiety than younger patients regardless of type of donation. Here, again there was less anxiety and depression among living donor transplanted patients (p<0.05). Another study reported similar results that patients older than 35 years old at the time of transplantation had more anxiety (p<0.05) than younger patients [14].

The difference in time on dialysis observed between the two studied groups was anticipated given the elective nature of living transplantation that allows less delay for transplantation. Patients with a history of dialysis more than 35 months had a significantly higher prevalence of depression than those with a history of dialysis lesser than 35 months. The prevalence of anxiety was not significantly associated with the time patients was on dialysis. Sign, *et al*, reported association between none of these variables. However, the study considered that depression was more common in patients with more prolonged dialysis [15].

It is reported that job status has an effect on the prevalence of depression [16] as it is occurred more in low socioeconomic people [17]. However, we could not find such relation for depression and anxiety. Alavi, *et al*, found similar results [4].

Like what Siyan, *et al*, [18] reported, we found that patients’ marital status did not affect the prevalence of psychotic disorders. This is in contrast to Akman’s findings who reported that depression was more common in single patients [19]. Generally, unmarried persons have more tendencies to be depressed [20]. This difference is probably due to greater support these patients receive by their family members.

There are contradicting data about association of psychotic problems and re-transplantation. In liver transplanted patients with a past history of transplantation, the prevalence of mental disorders was much higher than the first time transplantation [21]. However, we did not find such results which may be attributed to few number of re-transplanted patients in our series.

In conclusion, we found that psychological problems such as depression and anxiety are significantly higher in cadaveric renal recipients. Probably, periodic psychological evaluation should be recommended for kidney transplant recipients, especially for cadaveric group.
